# Acute effects of dynamic stretching combined with different blood flow restriction pressures on explosive performance in healthy young men

**DOI:** 10.7717/peerj.21614

**Published:** 2026-08-05

**Authors:** Peng Xin, Shiwei Jing, Meng Liu, Yan Huang, Kui Yan, Jun Ai

**Affiliations:** 1 College of Physical Education, University of Jinan, Jinan, Shandong, China; 2 China Institute of Football, Beijing Sport University, Beijing, China; 3 Research Department of Sports Science, Beijing Sport University, Beijing, China; 4 Beijing Shichahai Sports School, Beijing, China; 5 Shenzhen High School Experimental Campus, Shenzhen, Guangdong, China

**Keywords:** Blood flow restriction, Dynamic stretching, Explosive performance, Warm-up, Arterial occlusion pressure

## Abstract

**Objective:**

This study aimed to investigate the acute effects of dynamic stretching (DS) combined with different blood flow restriction (BFR) pressures on explosive performance in healthy young males, and to examine whether these effects differed across estimated arterial occlusion pressure (AOP) levels and performance tasks.

**Methods:**

In a randomized crossover design, 20 participants completed four warm-up protocols: DS alone, and DS combined with BFR at 30%, 50%, or 70% of estimated AOP applied to the proximal thighs. Explosive performance was assessed before and after each warm-up protocol using countermovement jump (CMJ), standing long jump (SLJ), 10-m sprint (10mSS), and 505 change-of-direction (505COD) tests.

**Results:**

CMJ showed a significant time × condition interaction. All DS+BFR protocols produced greater CMJ change scores than DS alone (all *p* < 0.01), with mean changes of 5.80 ± 1.99 cm, 5.95 ± 2.33 cm, and 5.15 ± 1.90 cm for the 30%, 50%, and 70% estimated AOP conditions, respectively, compared with 3.50 ± 2.21 cm in the DS condition. No significant differences were observed among the three BFR pressures. In contrast, SLJ, 10-m sprint, and 505COD showed no significant time × condition interactions, indicating that adding BFR did not provide statistically greater acute benefits than the DS condition for these tasks.

**Conclusion:**

Adding BFR to dynamic stretching produced an additional acute benefit for CMJ, but this effect was not pressure-dependent across the estimated AOP levels tested. The additional benefit of BFR was not observed for SLJ, 10-m sprint, or 505COD, suggesting that the acute effects of DS+BFR may be task-specific.

## Introduction

Warm-up is universally recognized as a fundamental and non-negotiable component of athletic preparation, serving as the critical bridge between rest and high-intensity performance. Its primary objectives encompass elevating core and muscle temperature, enhancing neuromuscular activation patterns, and psychologically priming athletes for subsequent exertion. These physiological and neural adaptations contribute to performance potentiation as well as injury risk mitigation (Sople & Wilcox, 2024; Shellock & Prentice, 1985; McGowan et al., 2015). Within this preparatory framework, the development and acute potentiation of explosive power emerge as particularly vital, given its status as a core determinant of athletic success. Explosive power is a core element of athletic performance, particularly crucial in movements requiring rapid and substantial force production such as jumping, sprinting, and changing direction (Aksović et al., 2020). Traditional dynamic stretching (DS) is widely adopted because it can increase muscle temperature, improve joint range of motion, and prepare participants for subsequent movement tasks (Iwata et al., 2019; McMillian et al., 2006). However, the acute performance benefits of dynamic stretching alone are not always consistent across explosive tasks, which has led researchers to explore complementary warm-up strategies (Opplert & Babault, 2018; Behm & Chaouachi, 2011).

Blood flow restriction (BFR) training involves applying external pressure proximally on a limb to partially restrict venous return while maintaining arterial inflow (Slysz, Stultz & Burr, 2016). BFR can create a localized hypoxic environment and increase metabolic stress during exercise, which may alter neuromuscular responses under low-load conditions (Scott et al., 2014). Recent studies using BFR combined with resistance-based conditioning activities suggest that BFR may contribute to acute performance-enhancing responses resembling post-activation performance enhancement (PAPE), while requiring lower external loads than traditional high-load conditioning activities (Zheng et al., 2022). However, whether similar acute effects occur when BFR is combined with dynamic stretching remains unclear.

Evidence regarding the acute effects of combining dynamic stretching with BFR (DS+BFR) on explosive performance remains limited, and the influence of different BFR pressure levels on warm-up efficacy has yet to be systematically examined. Different BFR pressures may influence the degree of vascular restriction and the practical tolerance of the warm-up protocol. However, because most existing evidence on BFR pressure has been derived from resistance-based or exercise protocols rather than dynamic stretching warm-ups, it remains unclear whether different estimated AOP pressures produce distinct acute effects when combined with dynamic stretching.

Dynamic stretching alone may increase muscle temperature and joint mobility, whereas BFR has been used as an adjunct to low-load exercise to modify the acute training stimulus (Suga et al., 2012). However, the physiological mechanisms by which BFR may modify exercise responses were not examined in the present study and should not be assumed to have occurred during dynamic stretching warm-ups. Therefore, the present study focused on whether adding BFR to dynamic stretching produces additional acute performance benefits, rather than on the physiological mechanisms underlying DS+BFR. This study aimed to investigate the acute effects of dynamic stretching combined with different levels of BFR pressure (30%, 50%, and 70% estimated AOP) on explosive performance in healthy young males. We hypothesized that adding BFR to dynamic stretching would produce greater acute improvements in CMJ compared with DS alone. We also explored whether any additional effects of BFR would extend to SLJ, 10-m sprint, and 505COD performance.

## Materials & Methods

### Participants

A total of 20 participants were recruited. Using a randomized crossover design, each participant completed all four warm-up protocols in a random order: (1) DS only, (2) DS + BFR-30% estimated AOP, (3) DS + BFR-50% estimated AOP, and (4) DS + BFR-70% estimated AOP. A 48-hour washout period was implemented between sessions to avoid carryover effects. Sample size estimation was performed using G*Power 3.1 for repeated-measures within-subject comparisons. Because directly comparable studies examining dynamic stretching combined with different BFR pressures were limited, a moderate effect size of 0.4 was selected *a priori* for planning purposes. With *α* = 0.05 and power = 0.80, the analysis indicated that a minimum of 16 participants was required. Therefore, 20 participants were recruited. Inclusion criteria were: (1) healthy young males aged 18–30 years; (2) no history of lower-limb musculoskeletal injury in the past 6 months; (3) no cardiovascular, metabolic, or neurological diseases. Exclusion criteria were: (1) any contraindication to blood flow restriction (*e.g.*, deep vein thrombosis, hypertension, peripheral vascular disease); (2) strenuous exercise within 24 h before testing; (3) use of ergogenic aids or medications affecting performance; (4) inability to complete all testing sessions. The study was approved by the Ethics Committee of Beijing Sport University (approval number: 2024098H), and all participants signed informed consent forms.

### Study design and procedures

**Figure 1:**
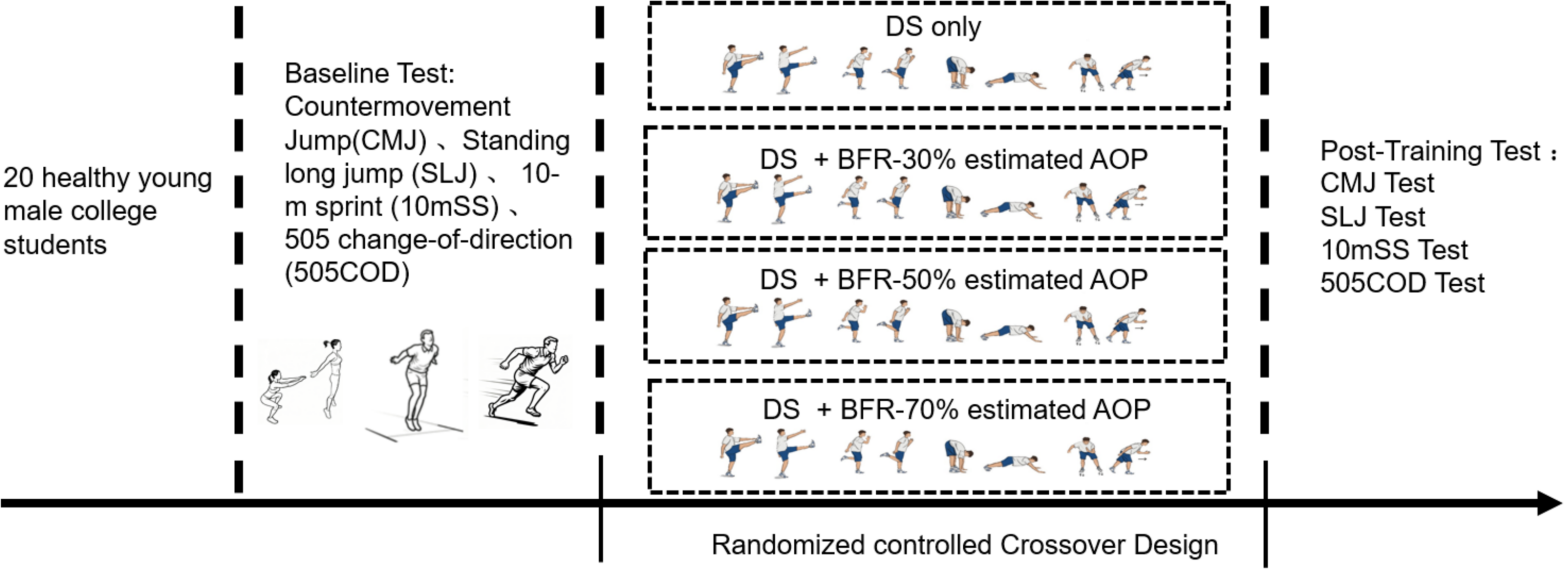
Study design and experimental flow. Study design flowchart. Twenty healthy young male college students completed four warm-up conditions in a randomized crossover order: dynamic stretching (DS) only, and DS combined with blood flow restriction at 30%, 50%, or 70% of arterial occlusion pressure (AOP). Baseline and post-warm-up tests included countermovement jump (CMJ), standing long jump (SLJ), 10-meter sprint (10mSS), and 505 change-of-direction (505COD) tests.

This study employed a randomized crossover design. Each of the 20 participants completed four warm-up sessions (DS, DS+BFR-30%, DS+BFR-50%, and DS+BFR-70%) in a randomized order, with a 48-hour washout period between each session to prevent residual effects. All tests were performed in a controlled laboratory environment (temperature: 22–24 °C, humidity: 50–60%) at the Sports Science Building of Beijing Sport University. To minimize expectancy bias, participants were informed during the informed consent process that the study aimed to compare different warm-up protocols, but they were not told any specific hypothesis regarding the effects of different BFR pressures. They were also not informed of the exact pressure values (30%, 50%, or 70% estimated AOP) they would receive during each session. All participants received standardized verbal instructions before each testing session, with no emphasis on any condition. The randomized crossover design helped balance potential order effects, and all performance tests were based on objective physical measurements (force plate, timing lights, tape measure). Testing sessions were conducted at the same time of day (between 2:00 PM and 4:00 PM) for each participant to control circadian effects (Fig. 1). Before each testing session, participants were instructed to refrain from strenuous exercise for 24 h, avoid caffeine and alcohol for 12 h, and abstain from food for at least 2 h.

Before the first testing session, resting systolic blood pressure (SBP) and thigh circumference were measured. Estimated arterial occlusion pressure (estimated AOP) was calculated using the method proposed by Tuncali et al. (2006): estimated AOP = (SBP + 10 mmHg)/KTP, where KTP represents the tissue padding coefficient selected according to thigh circumference. The BFR pressures were then prescribed as 30%, 50%, and 70% of each participant’s estimated AOP. Because AOP was estimated rather than directly measured using Doppler ultrasound or an automated limb occlusion system, the term “estimated AOP” is used throughout the manuscript. We used pneumatic cuffs (13 cm wide, KAATSU Master 2.0, Singapore) placed around the proximal part of both thighs, and the cuffs were kept inflated at the target pressure throughout the 10-minute warm-up.

### Warm-up protocols

**Figure 2:**
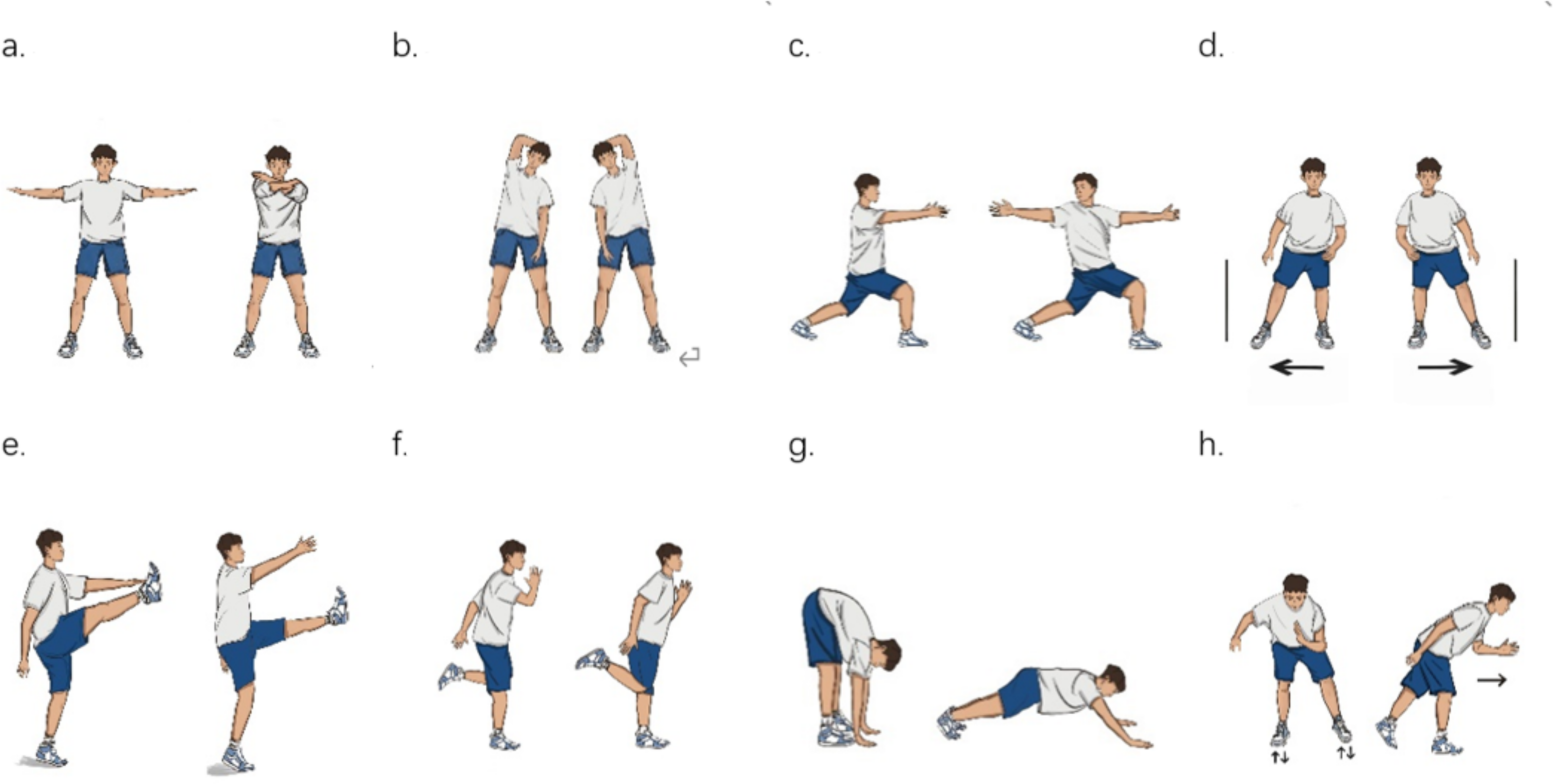
Dynamic stretching action. (A) Arm Crossovers; (B) Rotator Cuff stretch; (C) Walking Lunges with Trunk Rotation; (D) Lateral Shuffle; (E) Frankenstein Walk; (F) Heel-ups; (G) Inch worms (Hand Walk); (H) Neuromuscular Activation sprint initiation. Each movement was performed for 60 s during the 10-minute dynamic stretching routine.

Each session began with a 10-minute general dynamic stretching (DS) routine (Fig. 2 and Table 1), consisting of eight standardized movements: Arm Crossovers, Rotator Cuff Exercises, Walking Lunges with Trunk Rotation, Lateral Shuffle, Frankenstein Walk, Heel-ups, Inch Worms, and Neuromuscular Activation. Each movement was performed for 60 s with brief transitions.

**Table 1:** Name and specific requirements of dynamic stretching action.

Name of dynamic stretching action	Specific requirements
a. Arm Crossovers	Stand with feet shoulder-width apart and swing arms bilaterally across the chest while moving forward.
b. Rotator Cuff	Elevate the arm overhead with an elbow flexion and then gently lean to the side.
c. Walking Lunges with Trunk Rotation	Perform a large step forward while concurrently rotating arms horizontally.
d. Lateral Shuffle	Move laterally without crossing feet.
e. Frankenstein Walk	Extend hands in front of the body with palms down and kick toward the hands with legs extended.
f. Heel-ups	Kick heels toward the buttocks while moving forward.
g. Inch worms (Hand Walk)	Start in push-up position. Keep legs extended and walk toward the hands then move forward while keeping limbs stretched (repeated six times).
h. Neuromuscular Activation	Lower your weight, quickly step in place, and then sprint forward.

In the DS+BFR sessions, participants wore pneumatic cuffs around the proximal thighs during the dynamic stretching exercises. The cuffs were inflated to the designated pressure (30%, 50%, or 70% estimated AOP) and maintained throughout the 10-minute protocol. Proper cuff placement and pressure maintenance were ensured by experienced researchers, and participants’ subjective responses were continuously monitored for safety. In the DS-only session, the same dynamic stretching routine was performed without cuffs.

### Performance tests

Explosive performance was assessed before and immediately after each warm-up session using four tests. To ensure participants could perform at their maximal level and avoid the influence of fatigue, standardized rest intervals were implemented. Specifically, participants were provided with a 1-minute rest interval between consecutive attempts of the same test, and a 3-minute rest interval during the transition between different performance tests.

### Countermovement jump

Subject jump height was measured using a force plate (HAWKIN Dynamics, USA). Starting from an upright standing position with hands positioned on the hips to eliminate the influence of arm swing, subjects performed a rapid downward countermovement followed by an explosive maximal vertical jump upon verbal command. Three trials were completed, with the highest jump height (ICC = 0.98) recorded for subsequent analysis (Markovic et al., 2004).

### Standing long jump

Jumping distance was measured using a standardized tape measure. Subjects stood with toes positioned immediately behind the take offline. Following the start command, subjects executed a maximal forward jump utilizing an arm swing. Distance was measured from the take offline to the rearmost heel contact point upon landing, with subjects required to maintain balance upon ground contact (Marin-Jimenez et al., 2024). Three trials were performed, and the longest valid jump distance was used for analysis. This protocol demonstrates excellent test-retest reliability (ICC = 0.94) in adults.

### 10-meter sprint

Subjects adopted a standing start position with their dominant foot forward, positioned 0.5 m behind the start line and aligned such that the toe of the front foot was level with the start marker. On the start command, subjects initiated an all-out forward sprint without any preliminary backward movement. False starts were deemed invalid and necessitated a repeat trial (Franca et al., 2022). Two maximal effort trials were completed using a WITTY timing system (Microgate, USA), and the fastest time (ICC = 0.85) was used for analysis (Lockie et al., 2013).

### 505 change of direction

Subjects began in a stationary standing position at the start line. Upon the start command, they sprinted maximally to a line marked at 15 m. Using their dominant leg, subjects were required to place a single foot on or beyond the 15-m line, immediately execute a 180° turn, and sprint back 5 m to the finish line (Delaney et al., 2015). Total time was recorded using a WITTY timing system (Microgate, USA) with high test-retest reliability (ICC = 0.87). Two trials were performed, and the fastest completed time was retained for analysis.

### Statistical analysis

All statistical analyses were performed using IBM SPSS Statistics version 29.0 (IBM Corp., Armonk, NY, USA). Descriptive statistics were calculated for all variables and presented as means ± standard deviations (SD). Normality of distribution was assessed using the Shapiro–Wilk test. To examine the acute effects of different warm-up conditions on explosive performance, a two-way repeated measures analysis of variance (ANOVA) was conducted for each performance variable (CMJ, SLJ, 10 m sprint, and 505 change-of-direction), with condition (DS, DS+BFR-30%, DS+BFR-50%, DS+BFR-70%) and time (pre, post) as within-subject factors. Where significant interaction effects were detected, simple main effects and post-hoc pairwise comparisons were conducted using the Least Significant Difference (LSD) method. In addition, to further explore the specific effects of different BFR pressures, one-way repeated measures ANOVAs and polynomial trend analyses (linear and quadratic contrasts) were conducted on the change scores (post–pre) across the three BFR conditions (30%, 50%, and 70% of estimated AOP). This approach was utilized to directly assess how different warm-up protocols affected performance relative to the baseline, thereby detecting any potential linear or curvilinear pressure-dependent relationships. Effect sizes were calculated using Cohen’s d for pairwise comparisons and partial eta-squared (*η*^2^) for ANOVA results. Cohen’s d values were interpreted as follows: trivial (<0.2), small (0.2–0.6), moderate (0.6–1.2), and large (>1.2). A 95% confidence interval (CI) was reported for each effect size where applicable to provide precision estimates. Statistical significance was set at *p* < 0.05 for all tests.

## Results

The mean thigh circumference was 58.3 ± 3.8 cm, and the mean resting SBP was 119.8 ± 8.5 mmHg. The estimated AOP was 184.9  ± 13.9 mmHg. Accordingly, the prescribed cuff pressures were 55.5 ± 4.2 mmHg, 92.5 ± 7.0 mmHg, and 129.5 ± 9.7 mmHg for the 30%, 50%, and 70% estimated AOP conditions, respectively.

### Countermovement jump

CMJ performance demonstrated a significant time × condition interaction (*F* = 9.004, *p* < 0.001, *η*^2^ = 0.322). Both main effects for time (*F* = 223.35, *p* < 0.001, *η*^2^ = 0.922) and condition (*F* = 3.203, *p* = 0.030, *η*^2^ = 0.144) were also significant. To further explore this interaction, change scores (post-pre) were analyzed using a one-way repeated measures ANOVA, which confirmed a significant condition effect (*F* = 9.004, *p* < 0.001, *η*^2^ = 0.322). LSD *post-hoc* comparisons revealed that all three DS+BFR protocols produced significantly greater change scores than the DS condition (all *p* < 0.01), yet no significant differences were found among the three BFR pressures (all *p* > 0.05). Specifically, the mean change score was 3.50 ± 2.21 cm in the DS condition (*d* = 1.58 [0.90, 2.24]), compared with 5.90 ± 2.05 cm in DS+BFR-30% (*d* = 2.91 [1.96, 3.81]), 5.95 ± 2.33 cm in DS+BFR-50% (*d* = 2.55 [1.71, 3.35]), and 5.15 ± 1.90 cm in DS+BFR-70% (*d* = 2.71 [1.84, 3.53]). All change scores exceeded the SWC (1.58 cm), and no linear or quadratic pressure trend was observed.

### Standing long jump

For the standing long jump, statistical analysis yielded a non-significant time × condition interaction (*F* = 2.035, *p* = 0.119, *η*^2^ = 0.097). While the main effect of time was highly significant (*F* = 114.92, *p* < 0.001, *η*^2^ = 0.858), the main effect of condition was not (*F* = 0.419, *p* = 0.740, *η*^2^ = 0.022). Thus, although significant pre-to-post changes were observed in all conditions (all post > pre, *p* < 0.001), adding BFR did not confer a statistically greater improvement than DS alone. These change scores exceeded the SWC (0.045 m) for all conditions, presenting large effect sizes: DS (Δ = 0.089 m, *d* = 1.45 [0.81, 2.06]), BFR30 (Δ = 0.133 m, *d* = 1.23 [0.66, 1.78]), BFR50 (Δ = 0.125 m, *d* = 1.41 [0.78, 2.01]), and BFR70 (Δ = 0.152 m, *d* = 1.70 [0.99, 2.38]). No pressure-dependent trend was observed.

### 10-meter sprint

Regarding the 10-m sprint, the two-way ANOVA indicated no significant time × condition interaction (*F* = 0.744, *p* = 0.530, *η*^2^ = 0.038). However, significant main effects were observed for both time (*F* = 36.04, *p* < 0.001, *η*^2^ = 0.655) and condition (*F* = 5.71, *p* = 0.002, *η*^2^ = 0.231). *Post-hoc* comparisons for the condition effect showed that the DS+BFR-30% protocol resulted in significantly faster overall sprint times (pre- and post-average) than DS alone (*p* = 0.007), DS+BFR-50% (*p* = 0.019), and DS+BFR-70% (*p* = 0.004). Because the interaction was not significant, the improvements from pre to post did not differ significantly among conditions. Descriptively, DS+BFR-30% produced the largest mean reduction (Δ = −0.083 s, −4.6%), exceeding the smallest worthwhile change (SWC = 0.023 s) with a large effect size (Cohen’s *d* = 1.05, 95% CI [0.50–1.58]). The other conditions also showed likely beneficial improvements (DS: *d* = 0.60 [0.12, 1.07]; BFR50: *d* = 0.69 [0.20, 1.17]; BFR70: *d* = 0.67 [0.18, 1.15]), but none were statistically superior to DS in terms of the change score.

### 505 change of direction

**Figure 3:**
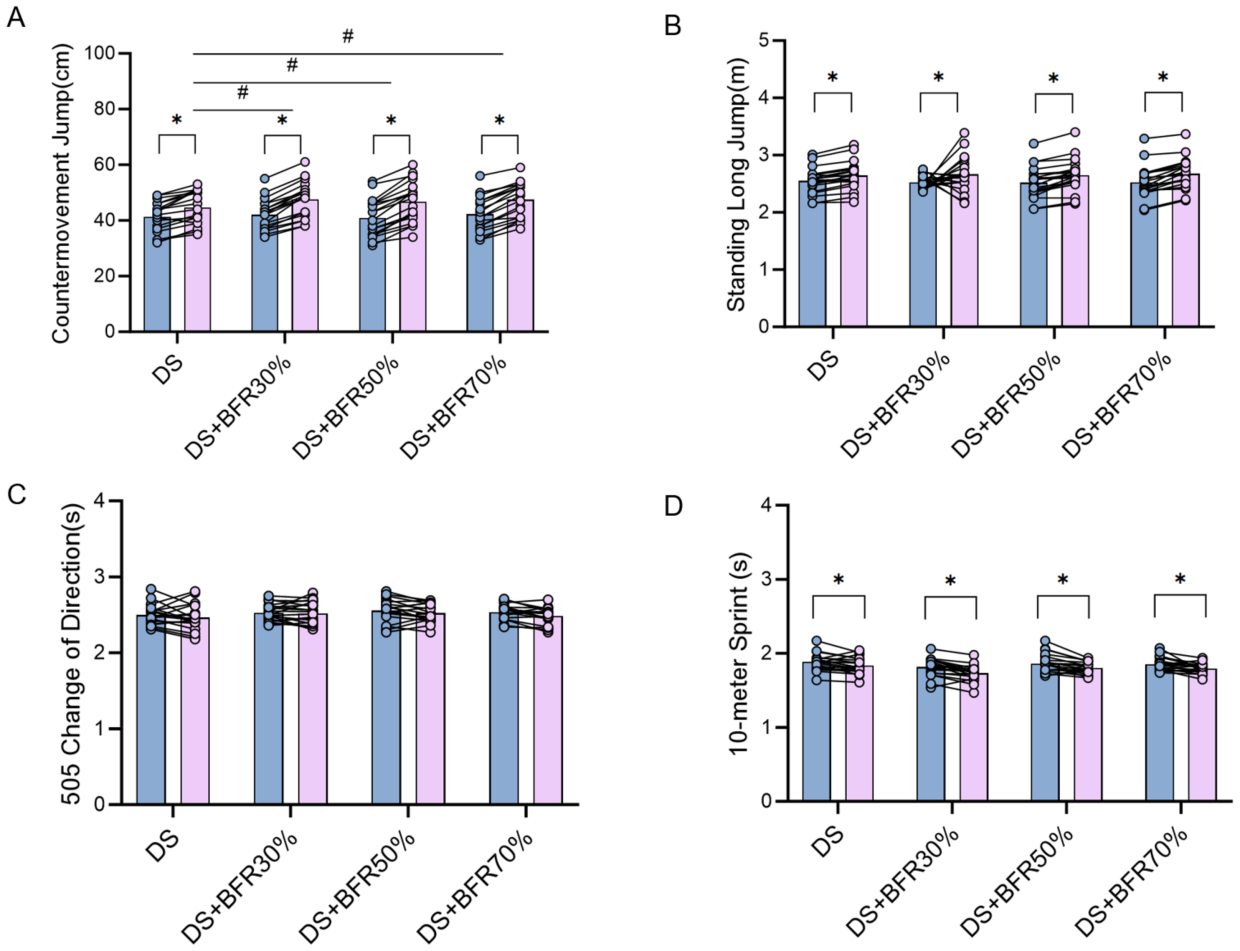
Horizontal performance before and after DS combined with DS+BFR and before and after DS. (A) Countermovement Jump; (B) Standing Long Jump; (C) 505 Change of Direction; (D) 10-meter Sprint. * indicates a significant difference from pre-test to post-test within the condition (main effect of time, *p* < 0.05). # indicates that the improvement in this condition is significantly greater than that in the DS-only condition (significant interaction effect, *p* < 0.01).

Finally, analysis of the 505 change-of-direction test revealed no significant time × condition interaction (*F* = 0.927, *p* = 0.434, *η*^2^ = 0.047). Furthermore, neither the main effect of time (*F* = 1.800, *p* = 0.196, *η*^2^ = 0.087) nor the main effect of condition (*F* = 2.350, *p* = 0.082, *η*^2^ = 0.110) reached statistical significance. Therefore, none of the warm-up protocols produced a statistically significant acute improvement in 505COD performance. Descriptive changes (Δ) and effect sizes are reported in Table 2 but given the lack of significant main or interaction effects, these represent non-significant fluctuations. Adding BFR did not provide any distinct benefit over DS alone (Table 2 Fig. 3).

**Table 2:** Changes in explosive performance following dynamic stretching warm-ups with and without blood flow restriction.

Condition	Pre (Mean ± SD)	Post (Mean ± SD)	Δ Mean Change	% Change	Cohen’s d
CMJ Height (cm)			SWC = 1.58 cm		
DS	43.05 ± 7.33	46.55 ± 8.09	3.50	8.1%	1.58
DS + BFR-30%	43.10 ± 7.27	48.90 ± 8.45	5.80	13.5%	2.91
DS + BFR-50%	42.05 ± 8.57	48.00 ± 8.97	5.95	14.1%	2.55
DS + BFR-70%	43.45 ± 8.28	48.60 ± 8.15	5.15	11.9%	2.71
Standing Long Jump (m)			SWC = 0.045 m		
DS	2.554 ± 0.224	2.643 ± 0.245	0.089	3.5%	1.45
DS + BFR-30%	2.530 ± 0.237	2.663 ± 0.308	0.133	5.3%	1.23
DS + BFR-50%	2.521 ± 0.281	2.646 ± 0.286	0.125	5.0%	1.41
DS + BFR-70%	2.524 ± 0.283	2.676 ± 0.270	0.152	6.0%	1.70
10-m Sprint (s)			SWC = 0.023 s		
DS	1.885 ± 0.113	1.840 ± 0.108	−0.045	−2.4%	0.60
DS + BFR-30%	1.816 ± 0.123	1.733 ± 0.107	−0.083	−4.6%	1.05
DS + BFR-50%	1.864 ± 0.131	1.801 ± 0.080	−0.063	−3.4%	0.69
DS + BFR-70%	1.856 ± 0.094	1.797 ± 0.069	−0.059	−3.2%	0.67
505 Change of Direction (s)			SWC = 0.027 s		
DS	2.494 ± 0.134	2.424 ± 0.253	−0.070	−2.8%	0.32
DS + BFR-30%	2.528 ± 0.112	2.521 ± 0.149	−0.007	−0.3%	0.07
DS + BFR-50%	2.558 ± 0.152	2.523 ± 0.120	−0.035	−1.4%	0.34
DS + BFR-70%	2.481 ± 0.210	2.474 ± 0.112	−0.007	−0.3%	0.04

**Notes.**

Δ = Post–Pre mean difference (positive values indicate improvement for jumps; negative values indicate improvement for sprint and COD tasks). SWC = 0.2 × between-subject SD from the DS pre-test. Cohen’s d thresholds: trivial < 0.2, small 0.2–0.6, moderate 0.6–1.2, large > 1.2. For CMJ (significant time × condition interaction), all BFR conditions improved significantly more than DS. The between-condition mean differences in change scores (BFR minus DS) and 95% CIs are as follows: BFR-30% = +2.35 cm [1.05, 3.65]; BFR-50% = +1.45 cm [0.37, 2.53]; BFR-70% = +2.05 cm [1.10, 3.00]. For SLJ, 10-m Sprint, and 505 COD, interactions were non-significant; therefore, between-condition differences in change scores are not statistically significant, and Δ/effect sizes are descriptive only. Without a no-warm-up control condition, the observed within-condition improvements cannot be solely attributed to the warm-up protocols, as repeated testing or time-related effects may contribute.

## Discussion

This study investigated the acute effects of DS combined with varying BFR pressures on explosive performance in healthy young males. The key findings are task-specific. For CMJ, incorporating BFR (30%, 50%, or 70% estimated AOP) into dynamic stretching significantly enhanced jump height compared to DS alone (all *p* < 0.01), with all three BFR pressures producing similarly large improvements (Δ = 4.6–5.8 cm, Cohen’s *d* = 2.55–2.91, exceeding the SWC of 1.58 cm). No significant differences were observed among the three BFR pressures, suggesting that the additional CMJ benefit did not differ across the 30%, 50%, and 70% estimated AOP conditions in this sample. For SLJ and 10-m sprint, although DS+BFR showed descriptively larger improvements than DS alone, the time condition interactions were not significant (*p* = 0.119 and *p* = 0.530), meaning that BFR did not provide statistically greater acute enhancement than DS alone. For 505COD, none of the warm-up protocols produced significant improvements (time main effect *p* = 0.196, interaction *p* = 0.434). Thus, the acute ergogenic effect of BFR during warm-up was not universal but was observed specifically in CMJ, a relatively constrained vertical jump task. These findings suggest that adding BFR at 30% estimated AOP may be considered when the goal is to acutely improve CMJ performance, although this interpretation should be made cautiously because AOP was estimated rather than directly measured; for the other explosive tasks, adding BFR did not provide statistically greater acute benefits than the DS condition.

Significant pre-to-post changes were observed in CMJ, SLJ, and 10-m sprint across the relevant conditions (all time main effects *p* < 0.001). For CMJ, the mean change score in the DS condition was smaller than those observed in the DS+BFR conditions (3.50 cm *vs.* 5.15–5.95 cm, *p* < 0.01). The mechanisms underlying the greater CMJ change scores observed in the DS+BFR conditions remain speculative. Previous studies using low-load resistance exercise with BFR have suggested that BFR may increase metabolic stress and alter neuromuscular responses (Suga et al., 2012). However, those findings were obtained from resistance exercise protocols and cannot be directly generalized to the present dynamic stretching warm-up protocol. Therefore, the present findings should be interpreted primarily as performance outcomes rather than direct evidence of a specific physiological mechanism. Nevertheless, for SLJ and 10-m sprint, although DS+BFR showed descriptively larger improvements, the time condition interactions were not significant (*p* = 0.119 and *p* = 0.530, respectively). These findings suggest that any additional effect of BFR may be task-specific, being observed for CMJ but not for SLJ, 10-m sprint, or 505COD in the present study. One possible explanation is that CMJ is a relatively constrained vertical task, whereas SLJ, sprinting, and change-of-direction tasks require greater horizontal force application, technique, and intermuscular coordination. However, because biomechanical and neuromuscular variables were not directly measured, this explanation remains speculative. Therefore, while BFR may provide an acute enhancement for certain explosive tasks, its superiority over DS alone should not be generalized without considering the specific movement characteristics.

For the 10-m sprint, descriptive statistics showed that DS+BFR-30% produced the largest mean improvement (Δ = −0.083 s, −4.6%, Cohen’s *d* = 1.05 [0.50, 1.58]), exceeding the SWC of 0.023 s. However, the time condition interaction was not significant (*p* = 0.530), indicating that the improvement from pre to post did not differ significantly among conditions in this study. Therefore, despite the descriptive advantage, the present data do not support the conclusion that BFR-30% is statistically superior to DS alone or to other BFR pressures in terms of change score. The significant condition main effect (*p* = 0.002) reflects differences in overall sprint times (pre- and post-average), with BFR-30% being faster on average, but this does not necessarily imply a greater acute enhancement. The lack of a significant interaction may reflect the limited sample size and the possibility that any true between-condition differences in change scores were small. Alternatively, sprint performance may depend more on acceleration technique and coordination than on the type of acute stimulus provided by BFR during dynamic stretching. In the present study, adding BFR did not produce a statistically greater pre-to-post change than the DS condition. Thus, based on the current results, we cannot claim that 30% estimated AOP is the optimal pressure for sprint enhancement. Although the mean change score in the DS condition was −0.045 s, adding BFR did not produce a statistically greater pre-to-post change in 10-m sprint performance than the DS condition.

The CMJ findings indicate that adding BFR to dynamic stretching produced an additional acute benefit over the DS condition, but this benefit did not differ among the 30%, 50%, and 70% estimated AOP conditions. Therefore, the present results do not indicate that higher BFR pressures produced consistently greater acute performance benefits across the outcomes assessed. From an acute warm-up perspective, the relevant question is not whether higher pressures maximize long-term adaptation, but whether increasing pressure provides additional short-term performance benefits. In the present study, higher estimated AOP pressures did not produce greater CMJ change scores than 30% estimated AOP. This suggests that, for CMJ in this acute DS+BFR context, 30% estimated AOP may be sufficient to obtain the observed additional benefit, although this should be interpreted cautiously because AOP was estimated rather than directly measured. The different responses between CMJ and the other tasks may be partly related to differences in task demands rather than the specific musculature affected by the cuffs. Although all tasks involved lower-limb muscles influenced by proximal thigh cuffs, CMJ is a relatively constrained vertical jump task, whereas SLJ, sprinting, and 505COD require greater horizontal force application, intermuscular coordination, trunk control, acceleration or deceleration mechanics, and movement technique. However, because biomechanical variables, neuromuscular activation, and muscle oxygenation were not directly measured, this task-specific interpretation should be considered speculative.

For the 505 change-of-direction test, neither the time main effect nor the time × condition interaction reached statistical significance. Therefore, based on the current data, we cannot conclude that any of the protocols, with or without BFR, produced a reliable acute improvement in 505COD performance. This lack of effect may be related to the complex nature of the 505 test, which involves deceleration, turning, and re-acceleration. Compared with CMJ, 505COD requires greater eccentric braking ability, trunk control, foot placement accuracy, and movement coordination (Philipp et al., 2024). Therefore, the acute stimulus provided by adding BFR to dynamic stretching may not be sufficient to produce a detectable additional benefit for this more complex task. However, because biomechanical and neuromuscular variables were not directly measured, this interpretation remains speculative. Overall, adding BFR did not provide a statistically greater benefit than the DS condition for 505COD in the present study.

In summary, this study suggests that the acute enhancement of explosive performance through BFR-augmented dynamic stretching may be task-specific but not pressure-dependent for the tasks where BFR appears beneficial. Specifically, for CMJ (a vertical jump relying on the stretch-shortening cycle), adding BFR significantly improves performance compared to DS alone, and 30% estimated AOP may be sufficient to obtain a similar additional CMJ benefit as higher estimated pressures in this acute DS+BFR context. For standing long jump, 10-m sprint, and 505 change-of-direction, BFR did not provide statistically greater acute enhancements than DS alone in the present sample, despite some descriptive improvements. Therefore, based on these findings, we cannot recommend the use of BFR for these tasks. Practitioners seeking to acutely enhance CMJ performance may consider incorporating BFR at 30% estimated AOP into a dynamic stretching warm-up, while the present findings do not support an additional benefit of BFR over the DS condition for SLJ, 10-m sprint, or 505COD. Future research should investigate the underlying mechanisms for the task-specific effects, explore potential long-term adaptations with repeated BFR warm-ups, and examine the influence of individual characteristics on the response to BFR.

This study has several limitations. First, the participants were healthy young males, limiting generalizability to females, adolescents, older adults, or elite athletes. Second, all testing occurred in a controlled laboratory; replication in real-world competition settings is needed. Third, only acute effects were assessed; the impact of repeated BFR warm-ups on long-term adaptations remains unknown. Fourth, the lack of significant interactions for SLJ, sprint, and COD may reflect the limited sample size and the possibility that any true between-condition differences in change scores were small. Fifth, the study did not include passive control condition (*e.g.*, no warm-up). Therefore, the observed changes cannot be definitively attributed to the warm-up interventions alone, as repeated testing or time effects may have played a role. Finally, physiological mechanisms were inferred from performance metrics. Future research should integrate electromyography and near-infrared spectroscopy to directly measure neuromuscular activation and local hemodynamics, providing deeper mechanistic insight.

## Conclusions

Dynamic stretching combined with BFR produced an additional acute benefit for CMJ, but not for SLJ, 10-m sprint, or 505COD. For CMJ, adding BFR at 30%, 50%, or 70% estimated AOP produced greater change scores than the DS condition, with no additional benefit from higher estimated pressures. In contrast, for standing long jump, 10-m sprint, and 505 change-of-direction, adding BFR did not confer statistically greater acute enhancements than the DS condition, despite some descriptive changes. These findings suggest that the additional effect of BFR during warm-up may depend on the specific task, and that 30% estimated AOP may be sufficient to obtain the observed additional CMJ benefit in this acute DS+BFR context. Accordingly, practitioners seeking to acutely improve CMJ performance may consider incorporating BFR at 30% estimated AOP into dynamic stretching, whereas the present findings do not support an additional benefit of BFR over the DS condition for SLJ, 10-m sprint, or 505COD.

## Supplemental Information

10.7717/peerj.21614/supp-1 Supplemental Information 1 Raw data anonymized, individual-level raw performance data for all 40 participants. It includes the pre- and post-warm-up measurements for the four explosive performance tests (Countermovement Jump height, Standing Long Jump distance, 505 Change-of-Direction time, and 10-meter sprint time) across all four experimental conditions (DS only, and DS combined with BFR at 30%, 50%, and 70% AOP).

10.7717/peerj.21614/supp-2 Supplemental Information 2 Participant characteristics and estimated AOP calculation data Participant characteristics, including age, height, body mass, thigh circumference, and resting systolic blood pressure, which were used to calculate estimated arterial occlusion pressure (estimated AOP) and prescribed BFR cuff pressures.

## Additional Information and Declarations

### Data Availability

The following information was supplied regarding data availability: The data is available in the Supplemental Files.
